# Design and synthesis of polymer nanoparticles with pH-responsive pan-HDAC inhibitor (C5) derived from norbornene block copolymers to increase C5 solubility and improve its targeted delivery to prostate cancer sites

**DOI:** 10.1080/14756366.2025.2530557

**Published:** 2025-07-23

**Authors:** Jacob Mathew, Anshul Mishra, Trong-Nghia Le, Jing-Ping Liou, Mei-Jung Lai, Vijayakameswara Rao Neralla

**Affiliations:** a Department of Chemical Engineering, National Taiwan University of Science and Technology, Taipei, Taiwan; b TMU Research Center of Drug Discovery, College of Pharmacy, Taipei Medical University, Taipei, Taiwan; c School of Pharmacy, College of Pharmacy, Taipei Medical University, Taipei, Taiwan; d Department of Chemistry, National Taiwan Normal University, Taipei, Taiwan; e TMU Research Center for Drug Discovery, Taipei Medical University, Taipei, Taiwan

**Keywords:** pan-HDAC inhibitors (HDACi), norbornene-derived block copolymer, hydrolysis, PC-3 prostate cancer cells

## Abstract

This study investigated the incorporation of C5, a pan-HDAC inhibitor, into a norbornene-derived block copolymer with pH-sensitive hydrolysis (PNEG-b-P(Nor-PABA-C5)) to generate NPs for prostate cancer treatment. Amphiphilic PNEG-b-P(Nor-PABA-C5) formed NPs in aqueous environments, with hydrophobic Nor-PABA-C5 monomers in the core and hydrophilic PNEG monomers on the surface. DLS analysis showed a particle size of 122 ± 12 nm with a PDI of 0.35, confirmed by SEM and TEM. TEM imaging revealed spherical morphology, enabling the NPs to transport hydrophobic pan-HDACi drugs to PC-3 tumour sites and facilitate release through hydrolysis under acidic conditions. The NPs exhibited pH-hydrolysis characteristics, with enhanced drug release (61 ± 1.7%) at pH 6.2 compared to pH 7.4 (35 ± 0.8%). MTT assay confirmed antiproliferative effect. Analysis of FITC/(PNEG-b-P(Nor-PABA-C5)) cellular uptake showed increased absorption in prostate tumours. Live/dead cell assays showed loss of viability, with increased red fluorescence and morphological disruption at higher concentrations over 48 and 72 h.

## GRAPHICAL ABSTRACT

**Figure:**
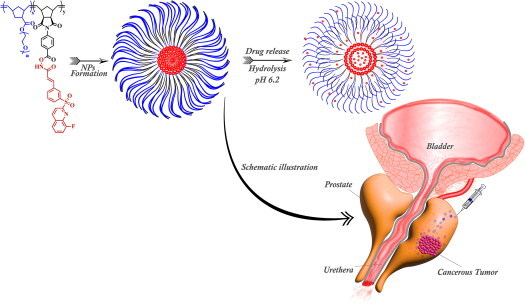

## Introduction

Histone inhibitors (HDAC*i*) are a class of epigenetic regulators that have garnered considerable interest as potential therapeutic agents. These inhibitors work by promoting the acetylation of histone and non-histone proteins, leading to a variety of effects on both tumour and non-tumour cells^1^^,^^2^. Cancer treatment has been revolutionised with the discovery of HDAC*i* that can alter the expression of epigenetically silenced genes, leading to cell cycle arrest, apoptosis, senescence, differentiation, and immunogenicity, as well as the inhibition of angiogenesis^3^. Moreover, these inhibitors have been shown to downregulate MYC expression in solid tumours and leukaemia^4^^,^^5^. When discussing the pharmacophore layout of HDAC*i*, it is essential to consider the structural properties, including the zinc-binding moiety, linker, and surface recognition moiety. The zinc-binding moiety is an integral structural feature of HDAC*i*, as it is responsible for interacting with the active site zinc ion in HDAC enzymes^2^. This interaction affects the enzymatic activity of HDAC enzymes, making it a key component in the inhibitory activity of HDAC*i*. The linker, another vital element, serves as a connecting domain within the pharmacophore layout. It navigates the pocket of the catalytic core, contributing to the inhibitory activity of HDAC*i*. The nature of the linker domain can vary among different classes of HDAC*i*, with examples including linear and aromatic linkers.The surface recognition moiety is further subdivided into a polar connecting unit and a hydrophobic cap, which plays an essential role in the interaction of HDAC*i* with histone and non-histone proteins^6^. This component impacts a wide range of effects in both tumour and non-tumour cells^7^. The hydrophobic cap contacts with the relay system surrounding the active site, contributing to the overall inhibitory activity of HDAC*i*. These structural features are crucial for the mechanism of action of HDAC*i*, as they enable the compounds to induce hyperacetylation in histone and non-histone proteins, leading to a broad range of effects in cellular processes^8^.

**Scheme 1.**
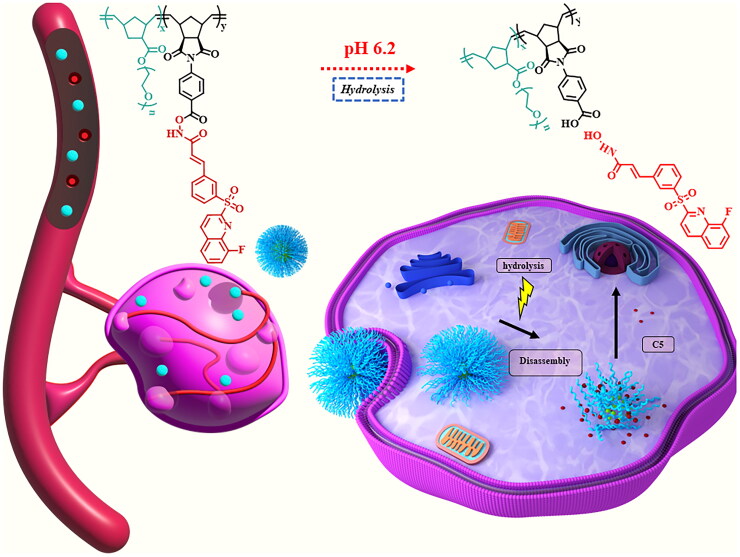
The diagram depicts an engineered polymer nanoparticle within the prostate cancer microenvironment

Despite significant progress in HDAC*i* drug development, challenges such as poor solubility, inadequate targeting, and inefficient delivery and controlled release persist. To address these obstacles, the development of HDAC*i*-based polymer nanomedicine presents a promising approach to enhance drug solubility, improve effective HDAC*i* drug delivery, and increase efficacy while mitigating adverse effects^1^^,^^9–11^. His research focuses on HDAC*i* drugs (2-(phenylsulfonyl) quinoline N-hydroxyacrylamides (8a–8k) developed by Lee et al. (compound 5 (C5)) as described in Scheme 1. This pan-HDAC*i* has exhibited selective potency against prostate cancer cells, demonstrating particular effectiveness against the PC-3 cell line^12^. The capacity of C5 to enhance the acetylation of these proteins in PC-3 cells suggests its potential to interfere with the epigenetic processes that are dysregulated in cancer cells, potentially leading to the inhibition of cell proliferation and the induction of cell death. Consequently, pan-HDAC*i* anticancer drugs with polymer backbones have the potential to exhibit increased efficacy and reduced side effects compared to traditional drugs. Successful development of pan-HDAC*i*-polymer drugs could significantly advance cancer treatment by providing more targeted and efficient therapies. This advancement could result in improved patient outcomes, reduced toxicity, and fewer adverse side effects compared to conventional treatments^10^^,^^13^.

In light of the above, we intend to develop a Norbornene derived PABA-C5 monomer (mono 1) and polyethylene glycol monomer (mono 2) derived from norbornene, which are described in detail in experimental section, and then copolymerise them using ring-opening metathesis polymerisation (ROMP) to yield block copolymers (PNEG-*b*-P(Nor-PABA-C5). Additionally, these conjugates demonstrate solubility in both water and biological medium, and also render C5 release via incubation in mild acid conditions possible due to their careful design. The pH elevation actually occurs due to the Warburg effect. This phenomenon involves cancer cells preferring aerobic glycolysis over oxidative phosphorylation, resulting in excess lactate and proton production that acidifies the surrounding environment. Additionally, tumours possess irregular vasculature, leading to oxygen deficiency and nutrient scarcity, which further promotes glycolysis and contributes to acidity. Consequently, a hydrolytic degradation under acidic condition was essential to facilitate the release of C5 at a mildly acidic pH level, similar to that found in cancerous cells^14–16^. Our designed linkages appear to be pH-responsive, suggesting that the C5 release from NPs of (PNEG-*b*-P(Nor-PABA-C5) is significantly accelerated at 6.2 pH, compared to 7.4. MTT experiments against PC-3 cancer cells demonstrate high anticancer activity for (PNEG-*b*-P(Nor-PABA-C5) NPs. The research results suggest that nanoparticles made from PNEG-*b*-P(Nor-PABA-C5) copolymers show promising capabilities as delivery vehicles for pan-HDAC*i* medications, featuring an acidic hydrolysis release mechanism.

## Materials and method

3-bromobenzenethiol, potassium carbonate (K_2_CO_3_), meta-Chloroperoxybenzoic acid, tert-butyl acrylate, Pd2(dba)3, Tri-tert-butylphosphonium tetrafluoroborate ([P(tBu)_3_] HBF_4_), N-Dicyclohexylmethylamine, Trifluoroacetic acid (TFA), Cis-5-Norborene-exo-carboxylic anhydride, Poly (ethylene glycol) monomethyl ether (*M*_n_ = 5000 g/mol), para-amino benzoic acid, acetic anhydride, sodium acetate, sodium bicarbonate. Second generation Grubb’s catalyst was purchased from AK Scientific, Inc. (CA, USA). 1,3 dicyclohexylcarbodiimde (DCC), 4-dimethylaminopyridene (DMAP), O-(Tetrahydro-2H-pyran-2-yl) hydroxylamine (NH_2_OTHP), tetrahydrofuran (THF), dichloromethane (DCM), 1-ethyl-3-carbodiimide hydrochloride (EDC.HCl), Hydrochloric acid (HCl), Methanol (MeOH), dimethyl formaldehyde (DMF), hydrochloric acid (HCl), diethyl ether (Et_2_O) Ethyl acetate, ethyl vinyl ether and pentane were purchased from Acros Organics.

### Cell studies

3-(4, 5-Dimethylthiazol-2-yl)-2, 5-diphenylterazolium bromide (MTT) was purchased from Sigma-Aldrich. Dulbecco’s modified Eagle’s medium (DMEM) and Opti-MEM foetal bovine serum (FBS) were purchased from Invitrogen Corporation (Carlsbad, CA).

Cell used in this study was PC-3 (a grade IV prostatic adenocarcinoma) from ATCC. (Catalogue: CRL-1435).

## Experimental sections

### Synthesis of compound 5

**Scheme 2.**
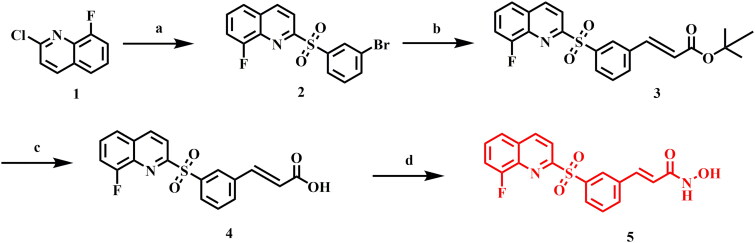
Reagents and conditions: (a) i. 3-bromobenzenethiol, K2CO3, DMF, 100 °C; ii. meta-Chloroperoxybenzoic acid, DCM, 0 °C to rt; (b) tert-butyl acrylate, Pd2(dba)3, [P(tBu)3] HBF4, N, N-Dicyclohexylmethylamine, DMF, 100 °C; (c) TFA, DCM, rt; (d) i. NH2OTHP, EDC.HCl, DMAP, DCM, rt; ii. 1 N HCl(aq), MeOH, 0 °C to rt

The synthesis of compound (5) involves a series of reactions as described in Scheme 2. The first step involves the reaction of 2-chloro-8-fluoroquinoline with 3-bromobenzenethiol under basic conditions, followed by oxidation with meta-Chloroperoxybenzoic acid to obtain intermediate **(2).** Subsequently, intermediate **(2)** undergoes Heck olefination with tert-butyl acrylate to yield intermediate **(3),** which is then hydrolysed with TFA to produce the corresponding carboxylic acid **(4).** Further, intermediate (**4**) is reacted with NH_2_OTHP in the presence of EDC.HCl, followed by hydrolysis with 10% TFA to produce the final compound **(5)**^12^. Detailed procedure was outlined in supporting information.

### Synthesis of norbornene-para amino benzoic acid (nor-PABA)

Cis-5-Norborene-exo-carboxylic anhydride (4 mmol) was charged into three necked flask and dissolved in 20 ml toluene followed by slow heating until it became clear solutions. To these solutions, para-amino benzoic acid (4 mmol) was added under regular stirring for fifteen minutes. Later, the temperature was turned off and the mixture was left to stir for 30 min. The reaction mixture was filtered before being vacuum-dried. The dry intermediate was then dissolved in DMF (30 ml) and heated to 50 degrees Celsius. Once the temperature was uniform, 2 ml of acetic anhydride (20.4 mmol) and 123 mg of sodium acetate (105.99 mmol) were added to the reaction mixture and stirred for 3 h. Later, the reaction mixture was introduced into 250 ml of water that had been acidified with 2.5 ml of HCl. The white substance was precipitated, filtered, washed multiple times in water, and dried under vacuum at elevated temperature to remove the water (yield = 86%). The synthesis of this compound was confirmed by ^1^H NMR and mass spectrometer in negative mode (Figure S1). Exact mass = 283.08, observed mass = 282.05

### Synthesis of nor-PABA-compound 5. (nor-PABA-C5)

The Nor-PABA was grafted with compound 5 (C5) using a simple esterification process. Here, the drug was connected to the Nor-PABA, forming an ester linker that was pH sensitive. Initially, Nor-PABA 90 mg (0.2 mmol), DCC 45 mg (0.2 mmol), and DMAP 2.6 mg (0.022 mmol) were added to a round bottom flask^17^. The mixture was dissolved in dimethyl formaldehyde in an inert environment and stirred for 30 min. C5 10 mg (0.268 mmol) was then put into a reaction condition and stirred for a whole day. Following the conclusion of the reaction, the product was put through filtering to remove the DCU residue. Ethyl acetate, sodium bicarbonate, and brine solution were later used to wash the reaction mixture. We used a vacuum to dry the final products. (yield = 85%). The synthesis of this compound was confirmed by ^1^H NMR and mass spectrometer in negative mode (Figure S2). Exact mass = 637.63, observed mass = 636.15

### Synthesis of nor-PEG

In accordance with our previous report^18^, Nor-PEG grafting was performed. In this process, PEG with a molecular weight of 5000 molecular weight was used. It was coupled using esterification and precipitated in diethyl ether with an 80% yield (Nor-PEG)

### Block polymerization of PNEG-b-P(nor-PABA-C5)

The block polymerisation of the PNEG-*b*-P(Nor-PABA-C5) was done by ring opening metathesis polymerisation (ROMP) method. A known of amount of Nor-PEG was transferred into a Schlenk flasks along with Grubb’s catalyst (15 mol%) under inter atmosphere. The resultant materials were dissolved in dichloromethane and methanol (9:1 v/v%) and kept for stirring for 4 h. Later, the Nor-PABA-C5 37.8 mg (0.084 mmol) was added to the flask and allowed to continue reaction for 24 h. The reaction was quenched in ethyl vinyl ether (20 µL) and later the product was precipitated using pentane^18^. The product was dried under vacuum condition and dissolved in THF and passed through neutral alumina to remove the catalyst and again precipitated and dried under vacuum. Gel permeation chromatography was done in DMF (1 ml/min). The molecular weight of the polymer was measured using PMMA standard.

### Preparation of PNEG-b-P(nor-PABA-C5) nanoparticle preparations

The polymer nanoparticle (NPs) was synthesised using a solvent precipitation technique. A specific quantity of polymer was dissolved in 2 ml of THF and introduced dropwise (1 ml/min) into 10 ml of water while stirring vigorously at 1200 rpm. The mixture was continuously stirred for 24 h, after which the solvent was eliminated through dialysis in deionised water^19^. Subsequently, the nanoparticles underwent lyophilisation. To analyse particle size, dynamic light scattering and TEM (Hitachi Ltd, Tokyo, Japan A Jasco) was employed.

### Assessing the surface change and pH -responsivity of the NPs

The drug release of PNEG-*b*-P(Nor-PABA-C5) NPs was measured for 72 h using dialysis. To summarise, the nanoparticles (5 mg/ml) have been introduced into the dialysis bag, immersed in 200 ml of release medium (7.4 and 6.2 pH level phosphate buffer solutions)^20^^,^^21^, revolving at 100 ± 5 rpm, and maintained at 37 °C. Aliquots of sample were collected at regular intervals, and the release medium was replaced with an equivalent amount of fresh PBS based on the pH value. UV-Visible spectroscopy was utilised to determine the drug content and amount released from the matrix. The stability of the NPs was evaluated using dynamic light scattering and TEM at two pH levels (7.4 and 6.2)^22^. The TEM images where later analysed using Image J software to determine the average size distribution in different pH levels. Negative staining was used analyse the TEM samples.

### Cytotoxicity of PNEG-b-P(nor-PABA-C5) NPs

The cytotoxicity of the nanoparticle was assessed using the MTT test. The cell line employed in the investigation was PC-3 human prostate cells^10^. PC-3 cells were sown in DMEM medium containing 10% FBS, 100 U mL^−1^ penicillin, and 100 μg mL^−1^ streptomycin in 24-well plates at a density of 1 cells per 2 × 10^4^ cells/well cultured overnight at 37 °C in a 5% CO_2_ incubator. The test material (PNEG-*b-*P (Nor-PABA-C5 NPs) was later added to each well at varying concentrations (10–200 µg) and incubated for 24 h. After incubation, 20 µl aliquots of MTT solution (5 mg/ml PBS) were applied to each well and incubated for 4 h. Once the media was removed the crystals were dissolved in 600 µl of DMSO. The absorbance was measured using a microplate reader at 570 nm^23^. The similar procedure was adapted for the L929 normal cell line for cross check compatibility. A similar technique was used to test cross-compatibility of the NPs with the L929 normal cell line^22^.

### Cell uptake of PNEG-b-P(nor-PABA-C5) NPs

The PC-3 cell was utilised to investigate the cellular uptake of PNEG-*b*-P(Nor-PABA-C5) NPs. By employing the nanoprecipitation method, FITC (1%, w/w) was wrapped into nanoparticles to produce FITC/PNEG-*b*-P(Nor-PABA-C5) NPs. Cells (1 × 10^4^ cells/well) were cultured in confocal dishes for 24 h, followed by the substitution of DMEM medium with fresh DMEM containing FITC/PNEG-*b*-P(Nor-PABA-C5) NPs (10 μg/mL) for incubation periods of 2, 4, 8, and 12 h, respectively. The cell uptake was then examined using fluorescence microscopy (Jasco spectrometer FP-8350) at the respective intervals.

### Live/dead assay

**Scheme 3.**
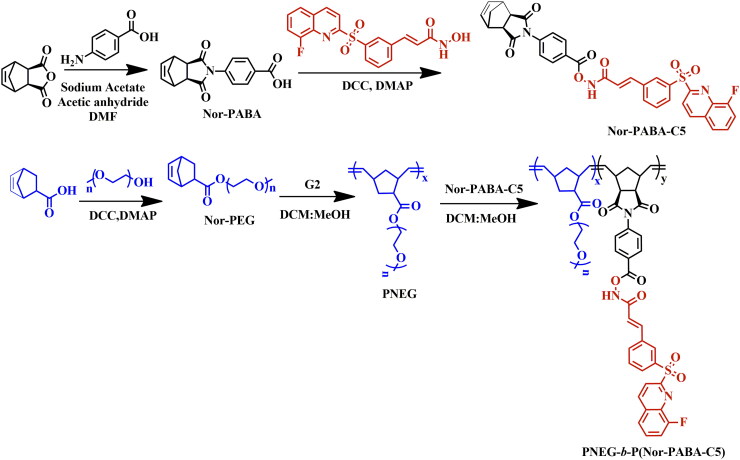
Synthesis scheme PNEG*-b-*P(Nor-PABA-C5).

The cells were culture as per cell uptake investigation and allowed to adhere for 24 h. Following, adherence, cells were treated with various NPs concertation for 24, 48 and 72 h. After treatment, the medium was removed and cells were washed with PBS. The assay utilised Calcein AM (1 mM/ml dmso), a cell-permeant dye that stains live cells green, and Propidium iodide (PI) (2 mg/ml), which marks dead cells red. After treatment, the cells were washed and stained with both dyes (Calcein AM- 2 µM, PI-5µg/ml) Qualitative images were captured using a fluorescence microscope 30 min after the dyes were added. The fluorescence signal was measured using an absorbance filter at emission/excitation wavelengths of 488/515 nm for live cells and 570/602 nm for dead cells (Scheme 3).

## Results and discussions

**Figure 1.**
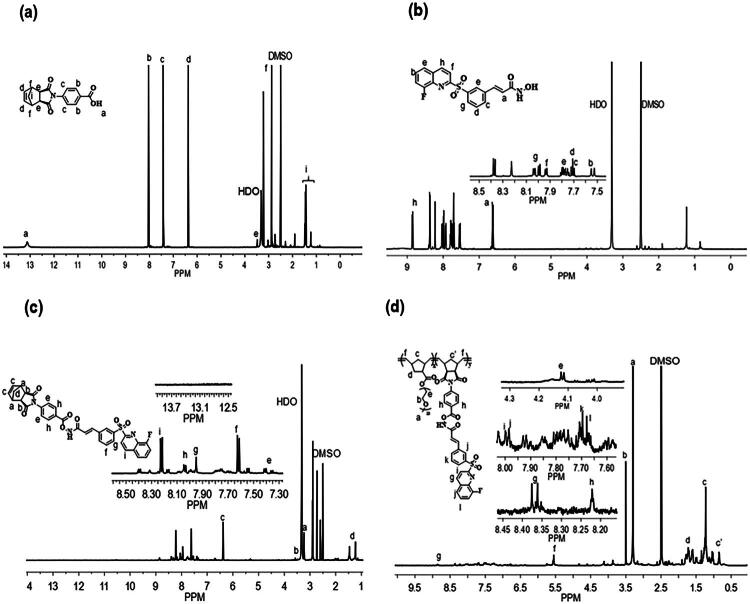
^1^H NMR studies (a) Nor-PABA, (b) Compound 5, (c) Nor-PABA-C5, (d) PNEG*-b-*P (Nor-PABA-C5).

In this study, we hypothesised that the site-sensitive characteristics of the drug-conjugated nanoparticles (NPs) would enable them to effectively target tumour sites via the EPR effect and subsequently release their payload due the pH responsive property. Furthermore, compound (C5) we grafted into the polymer, demonstrates a significant potential for cancer therapy. The C5 contains an N-hydroxyacrylamide group (C = C–CO–NH–OH), which is a key feature of HDAC*i.* To address solubility issues, a polymer nanoparticle drug system was developed for site-sensitive targeting. The site-sensitive polymer backbone was synthesised using the ROMP method. Nor-PABA was synthesised by combining Cis-5-Norborene-exo-carboxylic anhydride and 4-aminobenzoic acid in the presence of acetic anhydride and sodium acetate. Whereas Nor-PEG was designed as per the previous report^18^. The ^1^H NMR analysis (Figure 1(a)) revealed the carboxylic acid group at δ13.1 ppm and aromatic protons at δ 8.0–8.15 and 7.4–7.5 ppm. The exo-norbornene protons were observed at chemical shifts of δ 6.4, 3.5, and 2.9 ppm, whereas the norbornene-bridged hydrogen signals appeared at δ 1.55–1.71 ppm. Following the method described by Y. Ishii et al., C5 was linked to the carboxylic acid group of Nor-PABA using DCC DMAP. This approach resulted in the formation of a biodegradable ester bond, connecting the OH group of the HDAC isoenzymes to the COOH moiety^17^. The successful synthesis of Nor-PABA-C5 (mono 1) was confirmed by ^1^H NMR, which demonstrated the disappearance of the δ 13.1 ppm signal. Additionally, the aromatic and methylene shift of the drug were observed between δ 6.5–8.35 ppm (Figure 1(c)), indicating the successful grafting of mono 1. The ROMP technique was employed to continue the polymerisation of PNEG-*b*-P(Nor-PABA-C5).

Known amounts of monomers (mono 1 and 2) were weighed in two separate flasks under a nitrogen environment. The desired amount of second-generation Grubb’s catalyst was added and dissolved in a mixture of anhydrous dichloromethane and methanol (9:1 v/v%). Initially, mono 2 and Grubb’s catalyst were transferred to an inert atmosphere flask and dissolved in a suitable volume of a combinational solvent. The reaction mixture was stirred for four hours at room temperature. Then mono 1 was added to the flask and swirled under the identical circumstances as before for 24 h. The final reaction was evaluated using ^1^H NMR and GPC. The presence of PEG protons at δ 3.5 ppm suggests successful polymerisation of the two monomers (Figure 1(d)). The development of ethylene shifts at δ 5.7 ppm, as well as the elimination of olefinic protons (δ 6.4 ppm), confirmed the identity of PNEG-*b*-P(Nor-PABA-C5). The molecular weight of the finished product was measured using gel permutation chromatography using a PMMA standard. The DMF GPC had a Mn value of 18,047 and a polydispersity index (PDI) of 1.093 (Table 1). The Figure S3 demonstrates that a monodispersed polymer was synthesised utilising the ROMP approach with a second-generation Grubbs catalyst.

**Table 1.** Summarise data of block polymer.

Copolymer	^a^Mn (Dalton)	^a^Mw (Dalton)	PDI ^aMw/Mn^
PNEG*-b-*P(Nor-PABA-C5)	18 047	19 721	1.093

^a^ : Gel permeation chromatography.

### Characterisation of PNEG-b-P(nor-PABA-C5) NPs

**Figure 2.**
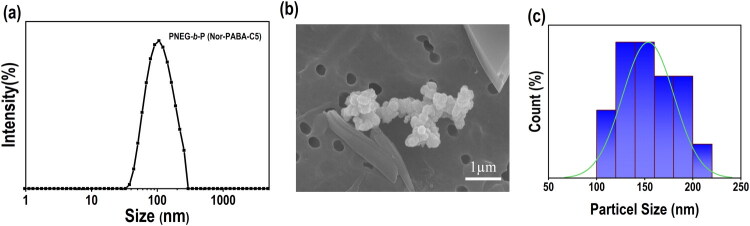
shows the particle and morphological examination of PNEG-b-P(Nor-PABA-C5) using SEM. (a) Particle size of the PNEG-*b*-P(Nor-PABA-C5) NPs by dynamic light scattering; (b) SEM image analysis (scale bar = 1 µm). (c) histogram analysis of PNEG-b-P(Nor-PABA-C5) NPs from SEM pictures using the Image J program.

Dynamic light scattering (DLS) was used to analyse the particle size (Figure 2). To achieve full solubilisation, a known quantity of PNEG-*b*-P(Nor-PABA-C5) NPs was dissolved in water and sonicated for a while. Aftermath, the aggregates were eliminated from the samples by filtering them through PTFE 0.45 µm. The NPs measured by DLS had an average size of around 122 ± 12 nm and a polydispersity index (PDI) of 0.35. Using SEM and TEM, the NPs’ morphology was ascertained (Figure 2(b,c) and Figure S5(a)). We determined from the pictures that the particles’ average size was between 120 and 135 nm using Image J software. The two- and three-dimensional analyses of the manufactured particles reveal that they were shaped like spheres and put together in a controlled way.

### In-vitro studies

Dynamics Light Scattering (DLS) was utilised to assess the nanoparticles’ (NPs) stability in two distinct pH ranges. The PNEG-*b*-P(Nor-PABA-C5) NPs exhibited substantial stability at pH 7.4 over a two-day period, as illustrated in Figure S4. However, under acidic conditions (pH 6.2), the particle size fluctuated significantly within the same time frame. The size variations were attributed to rapid hydrolysis, which altered the NPs’ stability after nine hours. Consequently, these findings suggest that the designed system demonstrates decent stability under normal physiological conditions.

The crosslinked nanoparticle’s morphology was examined using TEM at various pH levels (Figure S5). Under normal conditions, the nanoparticles showed no noticeable size alterations. However, at elevated pH, hydrolysis caused significant morphological changes. Previous research has indicated that successful hydrolysis enables the gradual release of pan-HDAC*i*, preserving its ability to inhibit tumour cell growth and enhance histone acetylation^24^^,^^25^. In acidic conditions, micelle stability may be compromised, potentially accelerating drug release. This mechanism is crucial for ensuring drug availability in the tumour microenvironment, where pH is typically lower than in healthy tissues, thus enhancing localised therapeutic effects.

The drug release characteristics of the developed material were analysed to determine release percentages under various pH conditions. The pan-HDAC*i* property drug was attached to the Nor-PABA monomer and polymerised using ROMP. UV-VIS spectroscopy quantified the drug content in the polymer backbone. PNEG-*b*-P(Nor-PABA-C5) NPs contained an average drug content of 3.543 ± 0.3%. Given that tumour microenvironments are typically acidic, a pH of 6.2 was selected to study drug release in tumour conditions^26^. A pH of 7.4 was considered physiological. A thorough analysis of the drug release profile revealed systematic C5 release during pH hydrolysis. This method allows C5 to avoid hydrolysis effects, which can result in rapid metabolic conversion, diminishing the novel compound’s anticancer properties. Additionally, rapid hydrolysis can reduce drug efficacy, especially in the bloodstream where various enzymes are present^27^. To address this issue, developing prodrug formulations like PNEG-*b*-P(Nor-PABA-C5) NPs is essential. These NPs shield C5 from hydrolysis, enabling sustained release over time.

**Figure 3.**
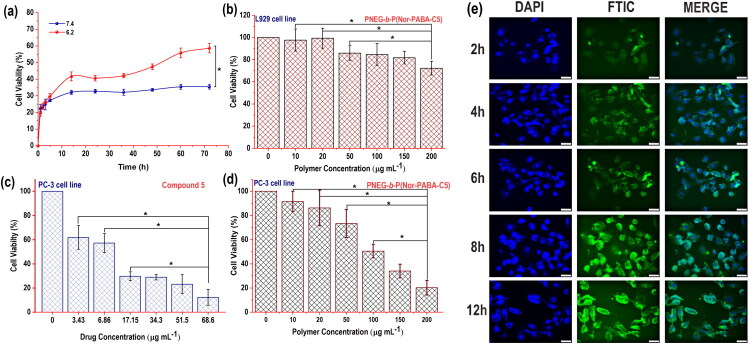
illustrates the *In-Vitro* studies of PNEG*-b-*P(Nor-PABA-C5). (a) depicts the cumulative drug release of the NPs under two different pH conditions. (b) illustrates the toxicity study in NPs in normal cell line (L929). (c) C5 towards PC-3 tumour cells (d) the cytotoxicity study of nanoparticle in PC-3 prostate cancer cell line. Values reported are the means ± SD for triplicate samples. *Indicate statistically significant differences at *p* < 0.05 (e) Cell uptake images of NPs towards PC-3 at different time periods (scale-50 µm).

The results shown in Figure 3(a) indicated that the process of hydrolysis allowed for the slow release of C5 from the nanoparticles (NPs), preserving its ability to suppress tumour cell growth and enhance histone acetylation. The stability of NPs may be compromised in acidic conditions, potentially leading to increased rates of drug release. Hydrolysis within the NPs can be accelerated by acidic environments, facilitating the liberation of C5. This mechanism plays a crucial role in ensuring drug availability within the tumour microenvironment, which typically has lower pH levels compared to normal tissues, thereby enhancing the localised therapeutic impact^25^.

The MTT assay was conducted using the PC-3 prostate cancer cell line. C5 with specific affinity for PC-3 cells was employed in this study. Previous research examined the regio-effect of the N-hydroxyacrylamide group on C5. C5 demonstrated a relatively high antiproliferative activity (GI_50_) against PC-3 cells, indicating its selective potency for this cell line compared to other models^12^. Despite these promising results, the drug’s hierarchical property cannot be fully exploited while maintaining its HDAC*i* characteristics due to solubility constraints. Therefore, a crucial element in drug delivery applications was the development of the polymer drug delivery system. The numerous intrinsic flaws in the chemotherapeutic medications are fixed by the designed NPs, which also increase their effectiveness. By enhancing the HDAC*i’* solubility and stability, the polymeric prodrug delivery method can extend blood circulation. Although C5 was previously reported, this engineered C5 (pan-HDAC*i*) with any polymer formulation had not yet been explored. Additionally, the created matrix’s site sensitivity can increase the pan-HDAC*i* drug’s pharmacokinetic characteristics and absorption, boost its accumulation within tumour sites, and lessen its systemic toxicity towards normal tissues. As shown in (Figure 3(b)), the relatively modest discharge of pan-HDAC*i* was attributed to the minimal hydrolysis properties of the NPs, which resulted in reduced toxicity to the normal cell line (L929). This effect was due to the different microenvironment compared to the tumour cells. Notably, cancer cells often have a more acidic extracellular environment than normal tissues, which can promote prodrug hydrolysis^28^.

The impact of polymer NPs on PC-3 was assessed using MTT over 24 h, with NPs concentrations from 10 to 200 µg/ml. The findings (Figure 3(d)) demonstrated significant toxicity in the cell line due to the subsequent pan-HDACi release. Data analysis revealed only 20% cell viability at 200 μg/ml concentrations, potentially resulting in substantial cell death. Figure 3(c) effectively demonstrates the dose-dependent effects of free C5 on PC-3 cells, showing a clear and consistent decline in cell viability as the concentration rises, which strongly confirms the drug’s potent antiproliferative properties. In sharp contrast, Figure 3(d) highlights the impressive effectiveness of C5 grafted nanoparticles (NPs) over a polymer concentration range of 10–200 μg/mL, equivalent to the C5 concentration. Remarkably, even at a lower C5 dose, these NPs achieve a similar reduction in cell viability to about 20% at the highest polymer dose. This compelling evidence highlights the nanoparticle system’s capability to enable efficient intracellular delivery and pH-triggered hydrolysis of C5, thereby maintaining its HDAC-inhibitory function. Together, these findings conclusively demonstrate that the NPs formulation not only retains C5’s therapeutic potency but also significantly boosts its effectiveness through controlled release and enhanced bioavailability at lower drug concentrations. This advancement signifies a transformative step in therapeutic delivery, offering improved outcomes with reduced dosages. Although C5 exhibited slightly higher toxicity towards PC-3 at higher concentrations compared to NPs. While the preceding document explores the effectiveness and mechanism of action of these compounds, it lacks specific information about their metabolic stability or transformation into inactive metabolites^12^. Additional research would be required to examine the metabolic pathways and stability of these compounds in biological systems.

## Discussions

Our team has devised a nanoparticles (NPs) system that administers therapy directly to the tumour site and displays antiproliferative properties against the PC-3 cancer cell line. Given the significant drawbacks associated with prostate cancer, it is imperative that research in this area be advanced. Towards this end, various nanomedical strategies have been implemented to enhance the efficacy of prostate cancer treatment^29^. For instance, Liu et al.^30^ reported on the development and evaluation of a novel pH-responsive dual-androgen-blocking magnetic molecularly imprinted polymer (FASC MIPs) for enhanced prostate cancer therapy. Moreover, Hu et al.^31^ examined polydopamine-based nanoparticles for synergistic chemotherapy of prostate cancer, while Raspantini et al.^29^ investigated PCL-*b*-TPGS polymeric nanoparticles. These studies either utilise conventional chemotherapeutic drugs such as DOX/docetaxel combined with immune regulatory small molecules for prostate cancer treatment. In recent years, a shift towards safer prostate cancer treatment methods has taken place due to the severe side effects associated with hormone therapy. Hormone therapy has been linked to adverse consequences such as osteoporosis and heart disease, which have prompted the search for alternative treatments like docetaxel. However, these alternative treatments also present their own set of side effects, including neuropathy and renal failure^32–34^. While the research on the application of HDAC*i* small molecules in combination with nano formulations is not yet comprehensive, some studies have explored this method.

For instance, a study authored by Y. Ishii et al.^17^ investigated the development and assessment of HDAC*i* prodrugs that aim to improve gene expression in prostate human cancer cells through the use of cationic nanoparticles (NPs). Although the article does not explicitly claim anticancer effects, modulating gene expression with HDAC*i* is a well-established area of research in cancer therapy. The enhanced gene expression observed in the study could be a step towards developing new anticancer treatments that target gene regulation pathways. In addition, Wang et al. conducted an *in-vitro* experiment to evaluate the efficacy of HDAC*i* NPs in comparison to small molecule HDAC*i* in enhancing the sensitivity of cancer cells to radiotherapy. They administered treatment with either NPs vorinostat or small molecule vorinostat followed by irradiation to PC-3 cells. The results from the clonogenic survival assay showed that NPs vorinostat was more effective than small molecule vorinostat in increasing the sensitivity of PC-3 cells to radiotherapy^35^.

Recent research has revealed a substantial relationship between prostate cancer and epigenetic mechanisms, particularly the role of HDAC*i*. These enzymes are involved in the removal of acetyl groups from histone proteins, a process that can lead to the compaction of chromatin and the silencing of gene expression^36^. HDAC*i* are crucial for normal cellular processes, but their dysregulation has been linked to several human cancers, including prostate cancer. The expression of HDAC*i* in prostate cancer has been observed to contribute to the proliferation and invasion of cancer cells. This has led to the development of HDAC*i* as potential anticancer agents^36^^,^^37^. Studies have shown that C5 inhibits multiple isoforms of HDAC without displaying selectivity towards any specific HDAC isozyme. This characteristic is referred to as being a “pan-HDAC inhibitor.” The study found that both C5 inhibited HDAC1, 2, and 6, which indicates that these compounds have a broad inhibitory effect on different HDAC isoforms (Table S1). In the context of PC-3 cells, the data suggests that compound C5, by virtue of its pan-HDAC inhibitory properties, can modulate the acetylation status of histone H3 and α-tubulin, which are proteins involved in the regulation of the cell cycle and apoptosis. The ability of C5 to increase the acetylation of these proteins in PC-3 cells suggests that it can interfere with the epigenetic processes that are dysregulated in cancer cells, potentially leading to the inhibition of cell proliferation and the induction of cell death. This is consistent with the observed antiproliferative activity of C5 against PC-3 cells (Table S1), as indicated by the GI_50_ value of 0.32 ± 0.04 µM^12^. The significance of intelligent nanocarrier systems is crucial for realising C5’s potential in a hierarchical manner, particularly in biomedical applications. When targeting tumour sites, site-sensitive nano-formulations are vital to minimise premature release and toxicity to healthy tissues. Additionally, due to the enhanced permeability and retention (EPR) effect of polymer systems, drug-loaded or conjugated nanoparticles can easily reach solid tumours and exhibit improved half-lives compared to hydrophobic drugs^38^. Furthermore, polymer systems can address the solubility issues of small molecules in water, leading to substantial advantages at the application level. The significance of PNEG-*b*-P (Nor-PABA-C5) should be recognised as a crucial contribution to the field of research. In contrast to previous PLGA nanoparticles^35^, our approach utilised the ROMP technique to engineer the polymer backbone and create uniform polymer units. In this instance, C5 was covalently linked to the polymer backbone, with its release specifically triggered in the acidic tumour microenvironment through hydrolysis under mildly acidic conditions. This resulted in a more secure drug release mechanism, as evidenced by in-vitro drug release studies^39^. These experiments showed minimal hydrolysis at pH 7.4, but a significant increase in release percentage at pH 6.2. Cytotoxicity tests indicated that the normal cell line (L929) had better viability compared to the PC-3 cell line, suggesting that premature drug release was not occurring and that hydrolysis was only active at the tumour site^40–42^. Compared to other HDACi, our PNEG-*b-*P(Nor-PABA-C5) NPs exhibited superior performance across all parameters. The average drug content in PNEG-*b-*P(Nor-PABA-C5) was higher than that reported for PLGA-based NPs by Wang et al., where vorinostat and quisinostat were loaded at approximately 2.2–2.3% w/w. The lipid-based K-182 prodrug developed by Ishii et al. incorporated 10 mol% of HDAC*i* prodrug relative to the total lipid, but lacked detailed weight-based loading data, limiting direct comparison. When comparing the release rates of PLGA-based systems with PNEG-*b*-P(Nor-PABA-C5), it was noted that the former exhibited a faster drug release, while C5 demonstrated a well-controlled release under acidic hydrolysis conditions. On the other hand, the K-182 lipid NPs relied on intercellular activation via ester or disulphide cleavage, resulting in a less predictable release profile. Furthermore, PNEG-*b*-P(Nor-PABA-C5) NPs showed potent, dose-dependent toxicity in PC-3 cells with good selectivity due to the “pan-HDAC inhibitor” characteristics. In contrast, the K-182 formulation demonstrated significantly higher toxicity at elevated concentrations, with only about 20% cell viability at NP-20K. These findings highlight the advantages of the norbornene-based copolymer, which reduces off-target effects.

Moreover, the research indicated that efficient cellular uptake was attributed to the drug’s specific characteristics and the amphiphilic polymer nanoparticle. ^43^^,^^44^. Additionally, the controlled molecular weight of PNEG-b-P(Nor-PABA-C5) achieved through the ROMP method enhances the EPR effect^45^, allowing the nanoparticles to effectively target the PC-3 tumour. This improves the cellular uptake ratio in cancer cells^43^^,^^44^. The spherical form of NPs contributes to their increased stability and prolonged circulation time. Due to their reduced surface energy, spherical NPs generally exhibit improved stability in biological fluids. Additionally, this shape enhances interactions with cell membranes, leading to better uptake at tumour sites and resulting in an increased cellular uptake ratio in cancer cells. Analysis of Figure 3(e) shows a significant increase in fluorescence intensity over time. These results indicate that the polymer nano formulation containing the pan-HDAC*i* exhibits enhanced therapeutic efficacy.

**Figure 4.**
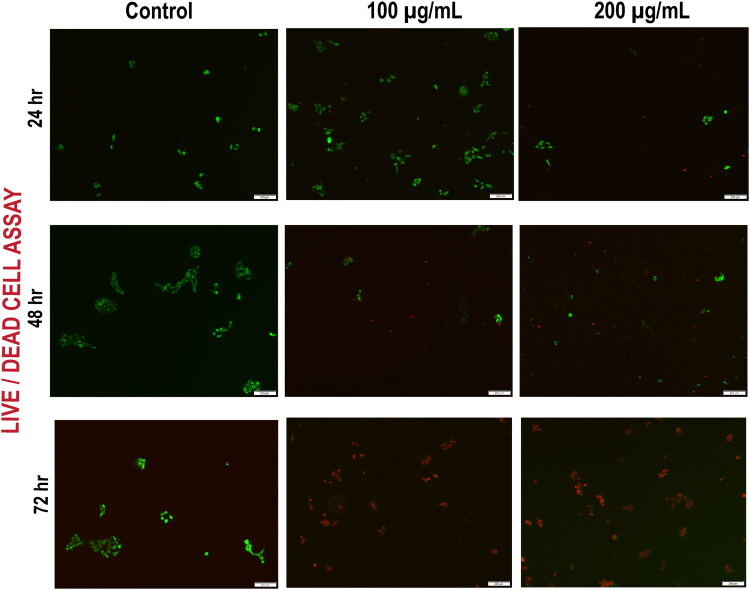
Live/dead cell assay of PC-3 cancer cell treated with NPs at varying concertation and time points. (Scale bar 200 µm).

Figure 4 presents the live/dead cell assay results for three treatment conditions (control, 100 µg/mL NPs, and 200 µg/mL NPs) across three-time intervals (24h, 48h, and 72h). Cells were treated according to the protocol outlined in the section. Calcein-AM served as a live cell marker, whereas propidium iodide (PI) was used to identify dead cells. The analysis revealed that in the control group, cells appeared bright green and well formed with minimal red fluorescence, indicating high viability at all time points. At 100 µg/mL NPs, there was a moderate decrease in viable (green) cells and a noticeable increase in red fluorescence, suggesting that early cytotoxic effects intensified over time. At the same time, an increased concentration (200 µg/mL NPs) resulted in irregular cell morphology with fewer live cells and more dead cells, indicating that the release of C5 in the tumour microenvironment increased the toxicity of PC-3 cancer cells. At 48 h post-treatment, cells exposed to 100 µg/mL NPs showed a moderate reduction in green fluorescence, indicating partial viability loss, along with a noticeable increase in red fluorescence intensity, suggesting apoptotic or necrotic cell death. Although many cells maintain membrane integrity and metabolic activity, morphological signs of cellular stress, such as rounding, shrinkage, and partial detachment, indicate early apoptotic progression in a subpopulation. In contrast, at 200 µg/mL, there was a near-complete loss of viable green-stained cells and predominance of red fluorescence, reflecting widespread membrane damage and irreversible cytotoxicity. The few remaining green-positive cells appeared highly distorted, fragmented, and structurally abnormal, implying that the green signal might persist transiently in metabolically active, but terminally compromised cells undergoing late-stage death. At 72 h, the control cells predominantly exhibited green fluorescence, indicating that most of the cells were viable. However, increased red fluorescence at 100 µg/mL and 200 µg/mL suggested that the compound induced moderate cell death over time. Additionally, morphological features such as cell shrinkage, disintegration, and apoptotic bodies were prominent. Some green fluorescence might persist in cell debris or dying cells, where the plasma membrane is not yet permeable to dead cell staining, but internal metabolic decline and morphological collapse have begun, as reflected in the 24- and 48-h studies. When integrating this conclusion with pan-HDAC*is*, HDAC inhibitors often induce programmed cell death, which takes hours, indicating early to mid-apoptosis where membrane integrity is not yet lost, but morphological distortions such as blebbing, shrinkage, or rounding are evident^46^. This trend was minimal in the 72-h observation, indicating the loss of membrane integrity.

In conclusion, our observations at various time points revealed that within the tumour microenvironment, C5 was released from the NPs matrix, resulting in the death of PC-3 cell lines. The sustained release profile of the NPs ensures a gradual intracellular accumulation of C5, leading to a time-dependent increase in cell death. This was clearly reflected in the live-dead cell assay, where cells began to die slowly at 24 h, entered apoptotic phases at 48 h, and underwent cell death at 72 h, particularly at higher concentrations. This mirrors the consistent drug action inherent to sustained release formulations.

## Conclusions

In conclusion, this study presents a novel approach to the conjugation of a pan-HDAC*i* anticancer drug within a polymer nanoparticle, enabling C5 to be released through hydrolysis in tumour environments. The PNEG-*b*-P(Nor-PABA-C5) NPs were synthesised utilising ring opening metathesis polymerisation (ROMP), employing a PEG backbone for the hydrophilic portion and Nor-PABA-C5 for the hydrophobic segment. This study showcases the simplest method for creating a block polymer with narrow molecular weight distribution. The polymer matrix formed spherical structures in aqueous solutions, as confirmed by various analytical techniques including DLS, SEM, and TEM. Studies showed improved affinity under normal physiological conditions (pH 7.4) in comparison to pH 6.2. The MTT assay verified toxicity against the PC-3 cell line, supporting the antiproliferative effects of C5, which result from the pan-HDAC*i* (C5) altering acetylation levels of histone H3 and α-tubulin, proteins essential for cell cycle control and apoptosis. C5’s ability to increase acetylation in PC-3 cells suggests its potential to interfere with cancer cell epigenetic mechanisms, possibly suppressing growth and inducing cell death. Furthermore, cell uptake analysis of FITC/(PNEG-*b*-P(Nor-PABA-C5)) at various time points indicates enhanced cellular uptake characteristics in prostate tumours. The live/dead cell assay reinforced these findings by progressive increase in red intensity (dead cells) over period of 72 h, particularly at higher concentration of NPs. Notably, live cells exhibited altered morphology and early signs of apoptosis at 48 h, followed by widespread cell death at 72 h, consistent with the delayed, sustained intracellular drug release profile. As a result, integrating C5 into a ROMP polymer backbone could offer a novel approach to optimise C5’s advantageous properties.

## Supplementary Material

supporting_information_anonymous_ Clean.docx

## Data Availability

The authors confirm that the data supporting the endings of this study are available within its ESI.
