# Supplementary material for: Design and synthesis of polymer nanoparticles with pH-responsive pan-HDAC inhibitor (C5) derived from norbornene block copolymers to increase C5 solubility and improve its targeted delivery to prostate cancer sites
Source: J Enzyme Inhib Med Chem. 2025 Jul 23;40(1):2530557. doi: 10.1080/14756366.2025.2530557 (PMC12288175; doi:10.1080/14756366.2025.2530557)
Supplement: supporting_information_anonymous_ Clean.docx [file IENZ_A_2530557_SM1284.docx]

**Design and synthesis of polymer nanoparticles with pH-responsive pan-HDAC inhibitor (C5) derived from norborene block copolymers to increase C5 solubility and improve its targeted delivery to prostate cancer sites.**

**Experimental Sections**

***Synthesis of 2-((3-bromophenyl) sulfonyl)-8-fluoroquinoline (2)***

A mixture of the 3-bromobenzenethiol (1.2 eq), K_2_CO_3_ (1.5 eq), and DMF was heated to 110 ˚C under N_2_ for 12 h. The resulting mixture was diluted with EtOAc and filtered. The filtrate was washed with H_2_O three times, and then the organic layer was purified through column chromatography. The resulting product (1eq) was dissolved in DCM, and then meta-chloroperoxybenzoic acid (2.1 eq) was added at 0 ˚C under N_2_ and the mixture was stirred at room temperature for additional 12 h. The reaction mixture was washed with cold 2N NaOH solution three times, and then the organic layer was collected and evaporated to provide product **2.** ^1^H NMR (300 MHz, DMSO-*d_6_*) δ 7.29-7.34 (m, 1H), 7.36 (d, 1H, *J* = 9.0 Hz), 7.40-7.56 (m, 4H), 7.58 (t, 1H, *J* = 1.8 Hz), 7.77-7.81 (m, 1H), 8.47-8.52 (m, 1H).

***Synthesis of tert-butyl (E)-3-(3-((8-fluoroquinolin-2-yl) sulfonyl) phenyl) acrylate (3)***

A mixture of intermediate **3** (1eq), *tert-butyl* acrylate (1.2 eq), Pd_2_(dba)_3_ (0.06 eq), [(n-Bu)_3_PH]BF_4_ (0.12 eq), N, N-Dicyclohexylmethylamine (1.1 eq), and DMF was heated to 100 ˚C under N_2_ for 12 h. The resulting mixture was diluted with EtOAc and filtered. The filtrate was washed with H_2_O three times and the organic layer was collected and purified by chromatography (ethyl acetate/hexane) to provide the corresponding acrylate. ^1^H NMR (300 MHz, DMSO-*d_6_*) δ 1.52 (s, 9H), 6.37 (d, 1H, *J* = 15.9 Hz), 7.14 (d, 1H, *J* = 9.0 Hz), 7.31-7.38 (m, 4H), 7.40 -7.48 (m, 2H), 7.53-7.62 (m, 2H), 8.13-8.17 (m, 1H).

***Synthesis of (E)-3-(3-((8-fluoroquinolin-2-yl) sulfonyl) phenyl) acrylic acid (4)***

To a stirred solution of intermediate **3** in DCM (10 mL), trifluoroacetic acid (3.5 mL) was added at room temperature and the mixture was stirred for 12 h. The solution was evaporated, and H_2_O was added. The pH value of the reaction mixture was adjusted to pH 4 by the addition of 2N NaOH and the precipitate was collected by filtration to afford the product **4**. ^1^H NMR (300 MHz, DMSO-*d_6_*) δ 6.57 (d, 1H, *J* = 16.2 Hz), 7.31-7.38 (m, 2H), 7.42-7.54 (m, 3H), 7.58-7.67 (m, 3H), 7.77-7.81 (m, 1H) 8.49 (dd, 1H, *J* = 1.8, 9.0 Hz), 12.44 (bs, 1H).

***Synthesis of (E)-3-(3-((8-fluoroquinolin-2-yl) sulfonyl) phenyl)-N-hydroxyacrylamide (5)***

A mixture of (E)-acrylic acid **(4)** (1 eq), NH_2_OTHP (1.2 eq), EDC.HCl (2 eq), DMAP (0.5 eq), and DCM was stirred at room temperature under N_2_ for 12 h. The reaction mixture was washed with H_2_O three times. The organic layer was collected and purified through column chromatography. The resulting product was dissolved in MeOH and then 1 N HCl solution (10-20 mL) was added slowly at 0 ˚C and the mixture was stirred at room temperature for 2 h. The reaction mixture was diluted with H_2_O and the solid was collected to provide the desired product **(5).[1]** ^1^H NMR (300 MHz, DMSO-*d_6_*) δ 6.48 (d, 1H, *J* = 15.9 Hz), 7.27-7.31 (m, 1H), 7.36 (d, 1H, *J* = 8.7 Hz), 7.45-7.54 (m, 6H), 7.77-7.81 (m, 1H), 8.47-8.51 (m, 1H), 9.05 (s, 1H), 10.73 (s, 1H). ^13^C NMR (75 MHz, DMSO-*_d6_*) 116.51, 116.75, 119.71, 122.81, 125.26, 125.32, 127.64, 130.07, 130.78, 130.88, 131.13, 131.38, 134.33, 137.17, 137.36, 140.00, 141.11, 141.15, 156.13, 158.56, 159.55, 162.98. HRMS-ESI calcd. for C_18_H_14_FN_2_O_4_S [M+H] 373.0658, found 373.0653.

**Cell studies**

The PC-3 cell were used as a representative model of castration-resistant prostate cancer (CRPC) due to their androgen receptor (AR)-negative status and metastatic origin. This model was chosen to evaluate the AR-independent therapeutic activity of C5, a HDAC*i* as previous studies have shown that C5 functions independently of AR signalling.[1]

**Table S1**: explain C5 antiproliferative activity (GI50) of and activities of HDAC isoforms 1,2, 6, and 8.[1]

| **Compound 5 (C5**) | | | | |
| --- | --- | --- | --- | --- |
| ^a^Antiproliferative  activity | **HL-60** | **HCT116** | **PC-3** | **A549** |
|  | 0.72±0.10 | 0.44 ± 0.11 | 0.32 ± 0.04 | 0.64 ± 0.04 |
| ^b^HDAC isoforms | **HDAC1** | **HDAC2** | **HDAC6** | **HDAC8** |
|  | 5.74 | 15 | 5.89 | 200 |

^a^GI_50_ ± SD (mM), ^b^IC_50_ ± SD (nM))

**Note: IC₅₀**: concentration causing 50% reduction in viability at endpoint.
**GI₅₀**: concentration causing 50% net growth inhibition from baseline.

**
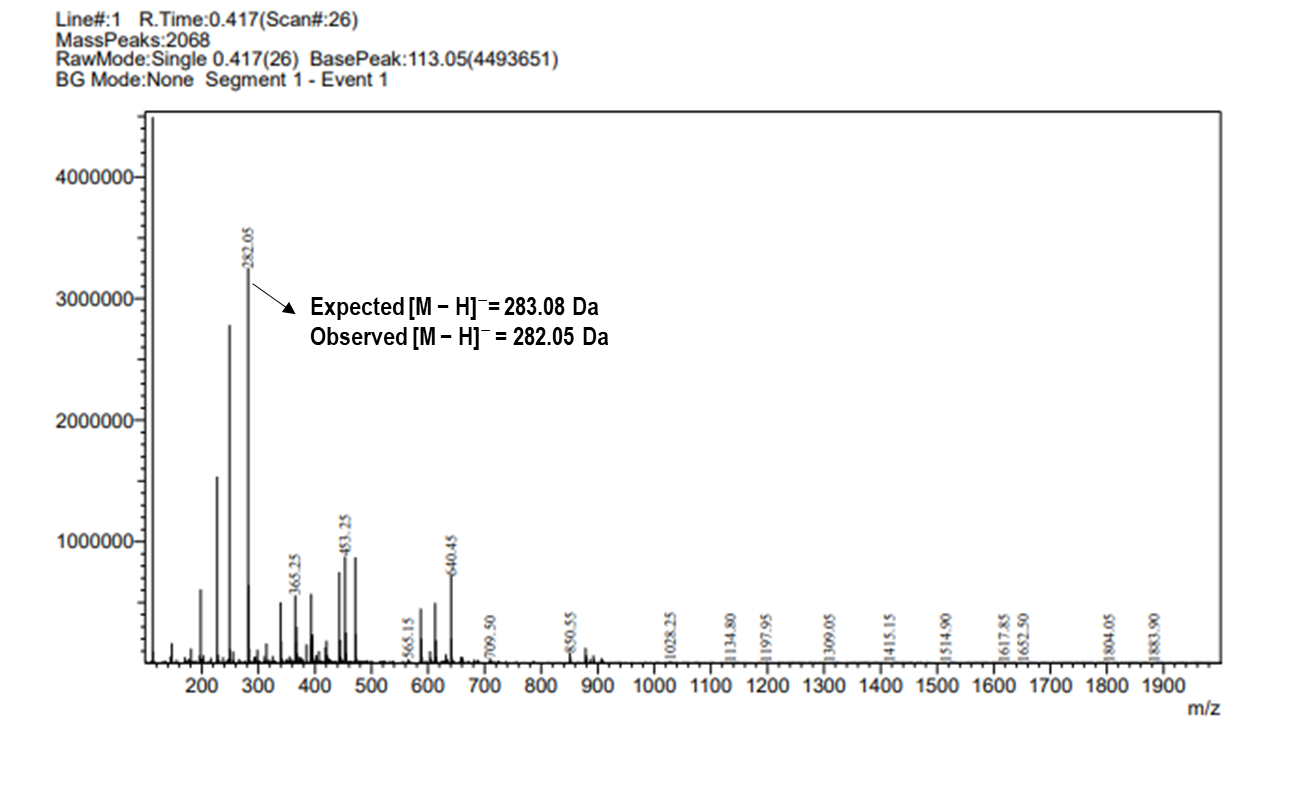
**

**Figure S1**: Mass spectrometry of Nor-PABA using Shimadzu instrument for the analysis of mass of desired compounds with ESI mode.

**
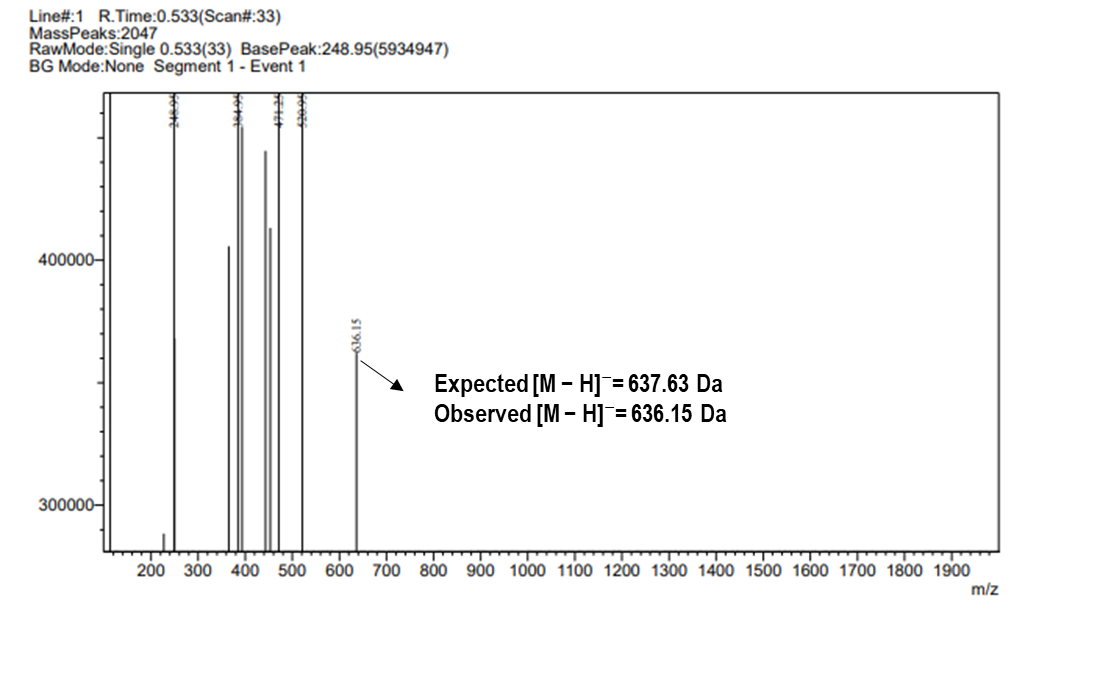
**

**Figure S2**: Mass spectrometry of Nor-PABA-C5 using Shimadzu instrument for the analysis of mass of desired compounds with ESI mode.


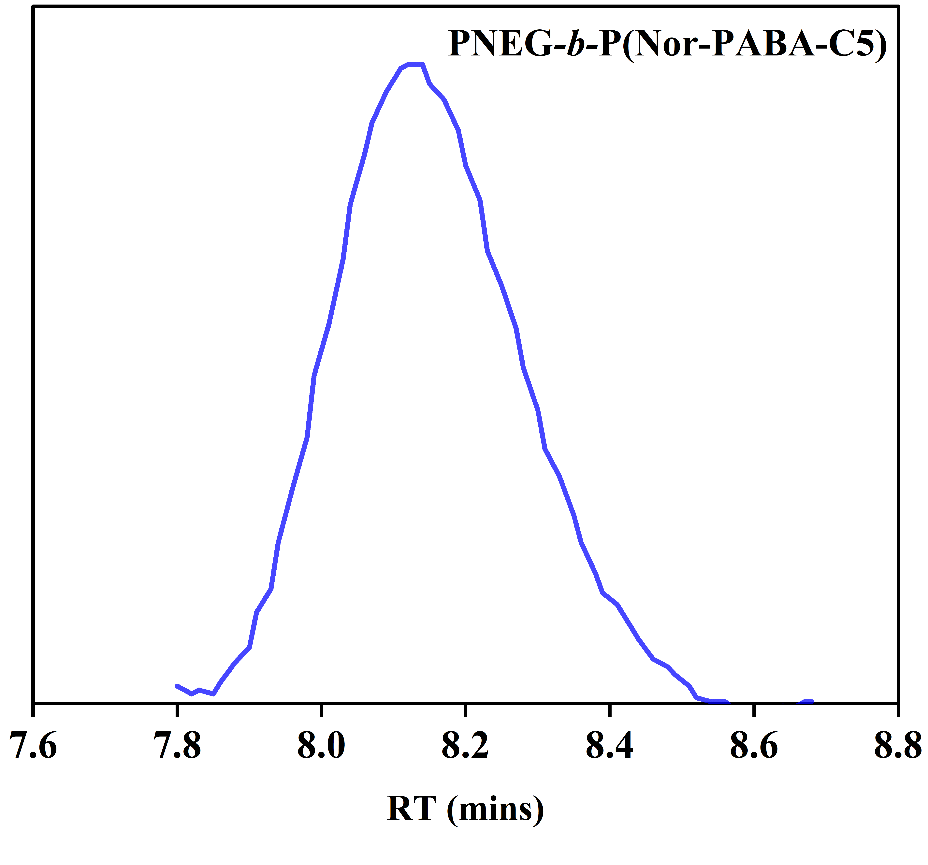


**Figure S3**: Molecular weight distribution of PNEG-*b*-P(Nor-PABA-C5) using polystyrene standard.


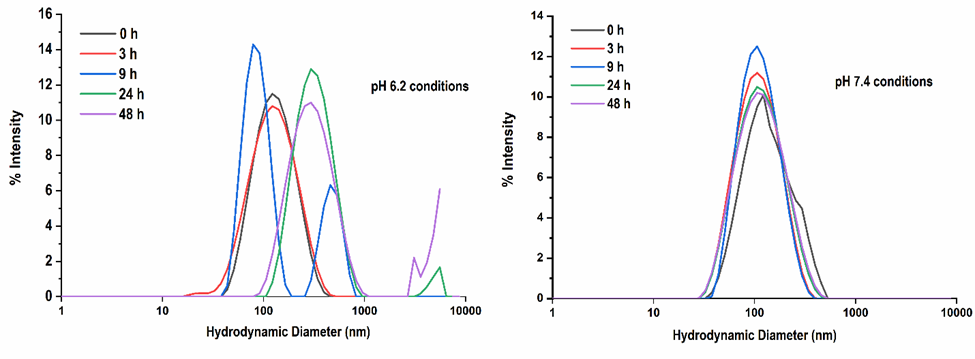


**Figure S4:** Particle size stability analysis in physiological & tumor environment. (a) under normal conditions (b) under tumor conditions in 6.2 pH.
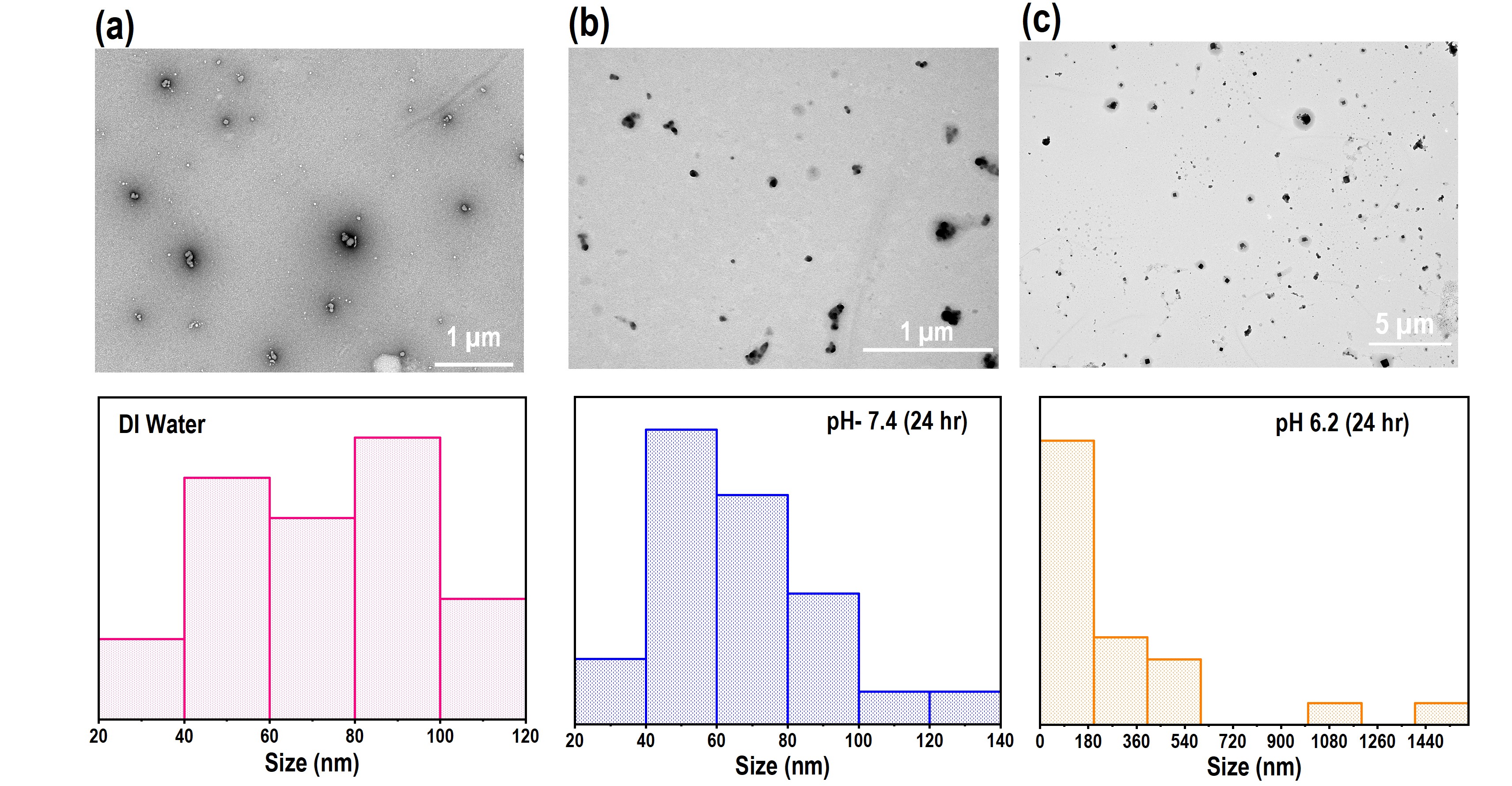
 **Figure S5**: Morphology, hydrolysis and size distribution analysis in different pH values


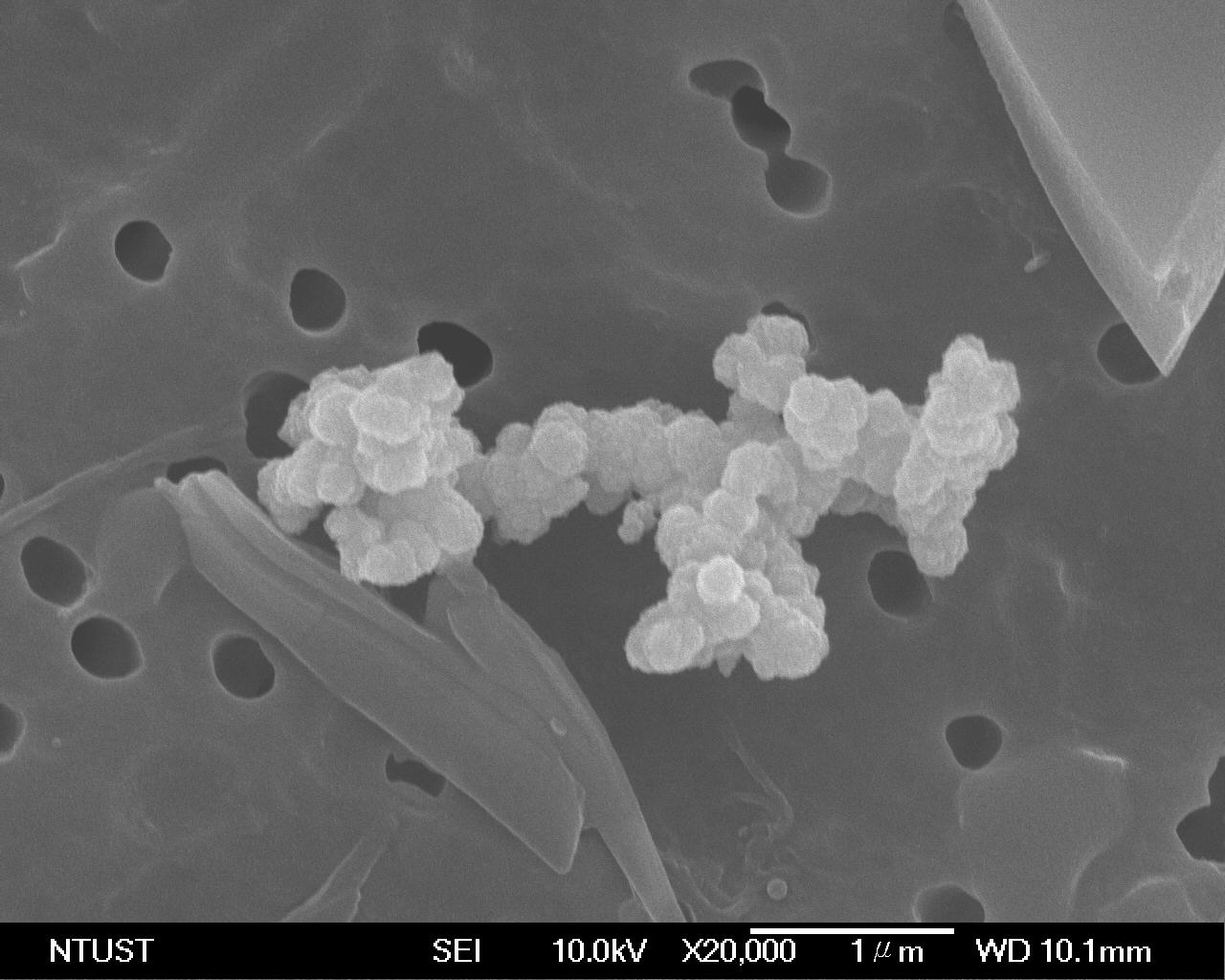


**Figure S6**: Individual uncropped, unprocessed image for each microscopic image. “Original Image for **Figure 2b.**


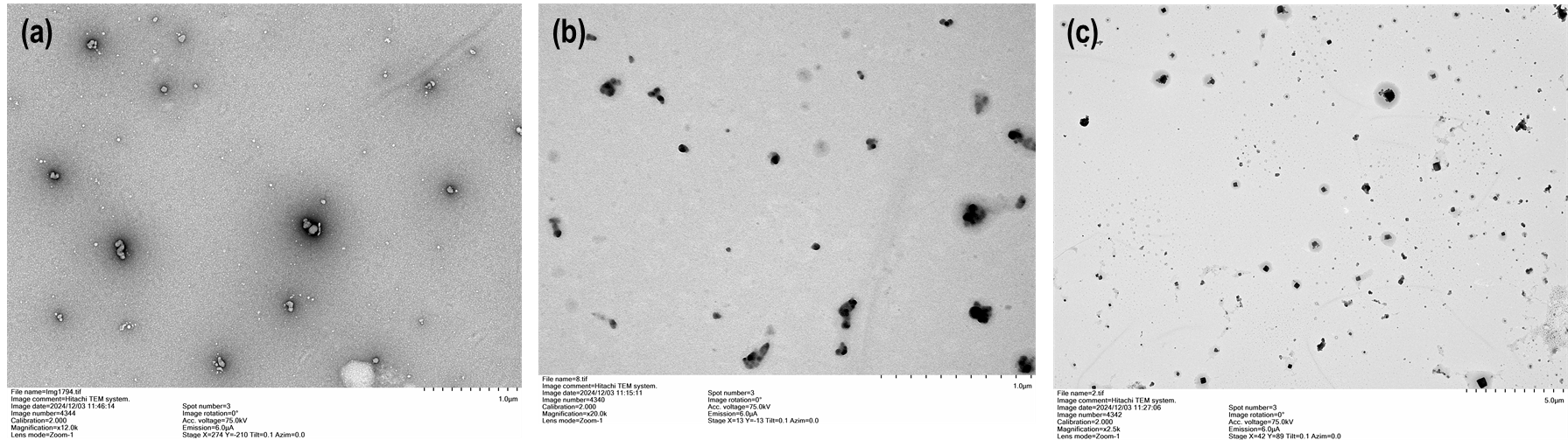


**Figure S7**: Individual uncropped, unprocessed image for each microscopic image. “Original Image for **Figure S5.**


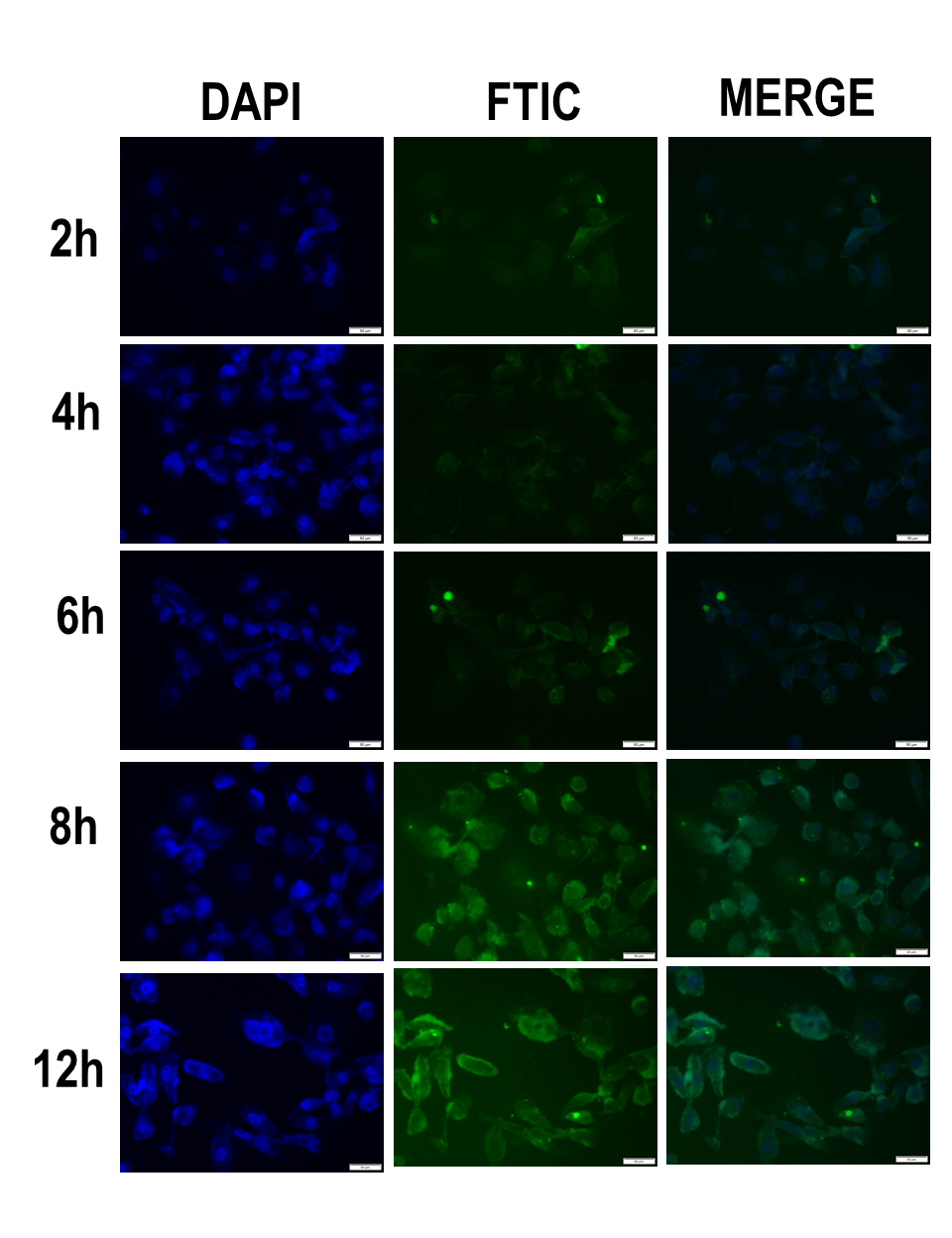


**Figure S8**: individual uncropped, unprocessed image for all microscopic images of cell uptake as per request. (Original image in main draft **Figure 3e**)


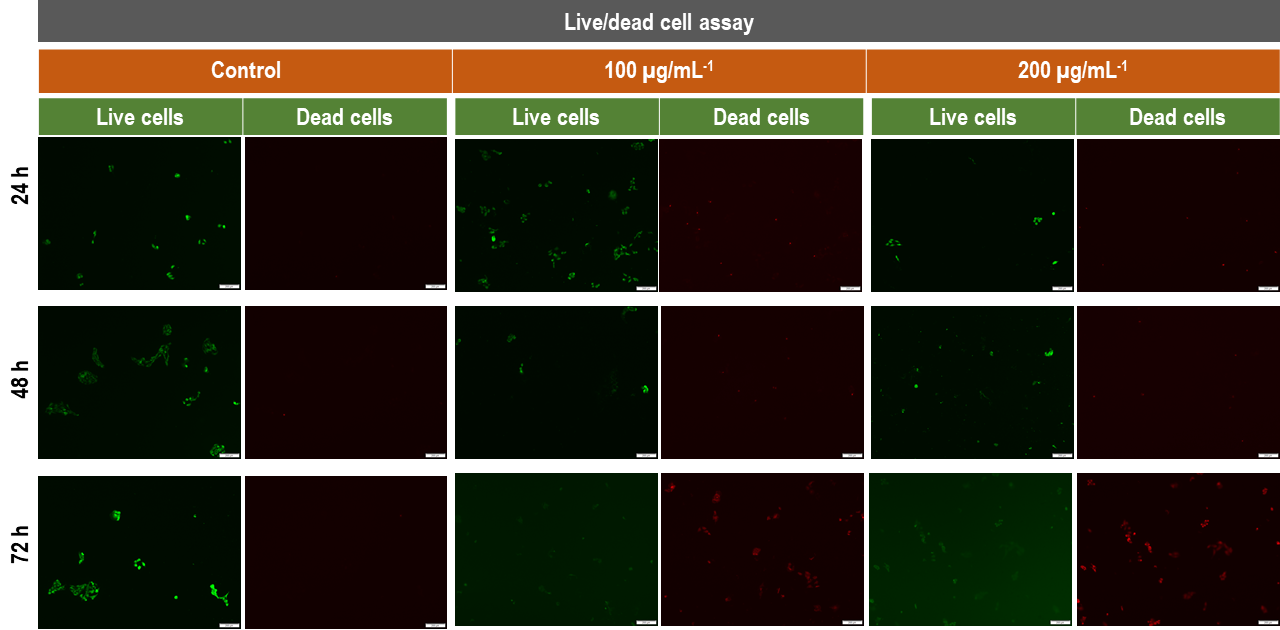


**Figure S9**: Individual uncropped, unprocessed image for each microscopic image. “Original Image for **Figure 4**

**Conflicts of interest**

There are no conflicts to declare.

**Data availability**

The authors confirm that the data supporting the endings of this study are available within its ESI.

**References**

1. Lee HY, Chang CY, Su CJ, et al. 2-(Phenylsulfonyl)quinoline N-hydroxyacrylamides as potent anticancer agents inhibiting histone deacetylase. Eur J Med Chem. 2016 Oct 21;122:92-101.
